# Predicting no return to sports after three months in patients with traumatic knee complaints in general practice by combining patient characteristics, trauma characteristics and knee complaints

**DOI:** 10.1080/13814788.2019.1646241

**Published:** 2019-08-21

**Authors:** Nynke M. Swart, Kim van Oudenaarde, Sita M. A. Bierma-Zeinstra, Johan L. Bloem, Patrick J. E. Bindels, Paul R. Algra, Monique Reijnierse, Pim A. J. Luijsterburg

**Affiliations:** a Department of General Practice, Erasmus MC, University Medical Center Rotterdam, Rotterdam, The Netherlands;; b Department of Radiology, Leiden University Medical Centre, Leiden, The Netherlands;; c Department of Orthopaedics, Erasmus MC, University Medical Center Rotterdam, Rotterdam, The Netherlands;; d Department of Radiology, Northwest Clinics, location Alkmaar, Alkmaar, The Netherlands

**Keywords:** Knee injuries, primary care, general practice, magnetic resonance imaging, return to sport

## Abstract

**Background:** It remains unclear to what extent patients with traumatic knee complaints aged 18–45 years seen in general practice experience difficulties with return to sports.

**Objectives:** This study aims to determine the proportion of patients with a knee trauma that return to sports at six weeks and three months follow-up. Also examined were associations between no return to sports and baseline patient/trauma characteristics, knee complaints and MR (magnetic resonance) findings, as well as the additive value of MR findings.

**Methods:** Included were patients with traumatic knee complaints participating in a randomized controlled trial assessing the cost-effectiveness of an MR scan in general practice. Patients were classified as ‘no return to sports’ or ‘return to sports’ (sports on pre-injury or adapted level). Potential baseline predictors for no return to sports were assessed using logistic regression analyses. The area under the curves (AUC) was compared.

**Results:** At six weeks and three months follow-up, 147 (59%) and 175 (74%) patients, respectively, reported return to sports. Combining patient characteristics, trauma characteristics and knee complaints predicted no return to sports with an AUC of 0.86 (95%CI: 0.81–0.90) at six weeks and of 0.82 (95%CI: 0.76–0.88) at three months follow-up. After adding MR findings, the AUC was 0.79 (95%CI: 0.71–0.87) at six weeks and 0.79 (95%CI: 0.70–0.88) at three months follow-up.

**Conclusion:** Three out of four patients with a knee trauma in general practice reported return to sports at three months follow-up. A combination of patient/trauma characteristics and knee complaints predicted no return to sports, whereas MR findings had no additive value.

**Trial registration:** Dutch trial registration: registration number: NTR3689. registration date: 7 November 2012.

KEY MESSAGES

- The odds of no return to sports at three months follow-up increases for patients who experienced trauma during sport, for patients with more pain at baseline and for patients who reported effusion at baseline.
- Magnetic resonance findings have no additive value in predicting return to sports.

## Introduction

A knee injury due to trauma during sports or leisure is a common indication for which patients visit their general practitioner (GP) [1]. Patients with traumatic knee complaints regularly ask when they can resume sports activities. To help address this, the GP’s tools for diagnosis and management of these complaints are described in the Dutch guideline for traumatic knee complaints [2]. In the acute phase, the diagnosis is mainly based on history taking, whereas physical examination adds little diagnostic value [3–5]. Studies have shown the potential diagnostic value of a magnetic resonance (MR) scan in traumatic knee complaints (requested by the GP) by improving patients knee-related quality of life and reducing medical costs [6–8]. In most patients with traumatic knee complaints in general practice, full recovery or significant improvement is reported after one year [9]. However, the return to sports after traumatic knee complaints remains precarious and most active young patients with traumatic knee complaints demand to return to sports as soon as possible. Currently, in patients aged 18–45 years visiting a GP, the impact of a knee trauma on their return to sports activities remains unclear. Therefore, this study aims to assess at six weeks and three months follow-up:

- the proportion of patients returning to sports after a knee trauma
- which patient characteristics, trauma characteristics, severity of knee complaints and MR findings, all measured at baseline, are associated with no return to sports
- whether MR findings have additive value in predicting no return to sports.

## Methods

### Design and setting

The present study included patients with traumatic knee complaints participating in a randomized controlled trial (RCT) that aimed to assess the (cost)effectiveness of an MR scan in general practice for patients with knee complaints due to trauma (TACKLE Trial) [10]. In the TACKLE trial, patients from 150 participating GPs were randomized to an MR scan or usual care. The recruitment for the TACKLE Trial took place from November 2012 to December 2015. The usual care group was treated according to the guideline of the Dutch College of General Practitioners for traumatic knee complaints, i.e. no MR scan [2]. The study was approved by the Medical Ethics Committee of the Erasmus Medical Center (Dutch Trial Registration: NTR3689) [11].

### Study population

Patients visiting their GP with knee complaints due to a trauma in the preceding six months were eligible for the TACKLE Trial. Patients had to be 18–45 years old; the restriction of 45 years was chosen to exclude patients with osteoarthritis as much as possible. Excluded from the study were patients with: (i) an indication for direct referral to an orthopaedic surgeon (e.g. fracture, locked knee or severe complaints after patella dislocation), (ii) knee complaints already treated in secondary care, (iii) previous surgical intervention of the affected knee, (iv) knee osteoarthritis diagnosed by a medical specialist, (v) other non-traumatic arthropathy (i.e. isolated patellofemoral joint pain), (vi) a previous MR scan for current knee complaints, or (vii) a contraindication for an MR scan. Furthermore, also excluded were patients: (i) who did not participate in sports before the knee trauma, and (ii) who did not return to sports after the knee trauma due to reasons other than knee complaints.

### Data collection and variables

*Questionnaire.* The following question about sports participation was included in the questionnaires filled in at baseline, and at six weeks and three months follow-up: ‘Are you able to participate in sports with your knee at this moment?’ The answers were categorized to: ‘yes, on the same level as before the knee trauma’, ‘yes, on an adapted level’, ‘no, not able to participate in sports because of the knee complaints’, ‘no, not able to participate in sports because of another reason’, and ‘not applicable, I do not do sport’. Afterwards, the answers were dichotomized to ‘no return to sports’ (not able to participate in sports because of the knee complaints) or ‘return to sports’ (sports at the same level as before the knee trauma, or at an adapted level).

*Baseline variables*. At baseline information on the following characteristics were collected: age, gender, height, weight, educational level (low/high), musculoskeletal comorbidity (yes/no) previous knee complaints (yes/no), symptom side (right/left), paid job (yes/no), and hours spent on the paid job per week. In addition, information on the date, occasion (sport/job/home/traffic/other) and the mechanism (fall/rotation/bump/squatting) of the knee trauma were assessed and dichotomized to: trauma during sport (yes/no) and rotational trauma (yes/no). In addition, the following were also assessed: the type of sport (ball sport: yes/no), hours of sport per week, and whether the sport was played in competition before knee trauma (yes/no).

*Baseline scores of outcome measures*. The baseline scores of the following outcome measures were used to assess the severity of knee complaints:

- The numeric pain rating scale (NPRS; scores ranging from 0 = no pain, to 10 = unbearable pain), for the average severity of knee pain during the previous 48 h and the previous week [12].
- The Lysholm scale (primary outcome measure of the TACKLE Trial) comprising 8 items on symptoms and limitations in activities (scores ranging from 0 to 100, with higher scores indicating better knee function [13].
- A modified Tegnér score to measure workload and sport participation, ranging from 0 = not able to work/sport due to knee complaints, to 10 = complete return to work/sports [13].
- The five dimensions of the knee injury and osteoarthritis outcome score (KOOS) to measure disability due to knee complaints [14]; the KOOS consists of five dimensions (pain, symptoms, function in daily living, function in sport and recreation, and knee-related quality of life) rated on a scale from 0 to 4: for every dimension, a score is calculated on a scale from 0 to 100 with a higher score indicating better knee function.
- The shortened version of the Tampa scale that measures fear of pain, movement and injury (TSK-11), scored from 1 = strongly disagree, to 4 = strongly agree [15,16]; the total score ranges from 11 to 44, with a higher score indicating more fear regarding pain, movement and injury.

*MR findings*. MR findings were scored by one of the 12 participating (experienced) radiologists at a median of 13 (interquartile range; IQR: 8–20) days after inclusion. The following items were scored: the amount of synovial fluid (effusion), abnormalities in soft tissues, meniscal injuries, anterior and posterior cruciate ligament ruptures, medial and lateral collateral ligament distortions and bone and cartilage injuries. The MR findings were dichotomized to the presence or absence of effusion, a bone bruise of the femorotibial joint (FTJ), fracture, traumatic meniscal tear (longitudinal, radial or complex meniscal tear), grade I–III distortion of the medial or lateral collateral ligament (MCL/LCL), partial or complete anterior or posterior cruciate ligament tear (ACL/PCL) and cartilage damage grade I–IV.

### Statistical analyses

Descriptive statistics were used to describe the participants. Data were tested on a normal distribution with the Kolmogorov–Smirnov test. The mean and standard deviation (SD) were reported in case of normal distributed data and median and IQR in case of skewed data. The baseline associations of patient characteristics, trauma characteristics, severity of knee complaints and MR findings with return to sports (1 = no, 0 = yes) were assessed with logistic regression analyses, adjusted for the time from trauma to study inclusion and return to sports at baseline. Candidate predictors for the logistic regression analyses were selected based on expert consensus (PL, SBZ, NS). The number of selected candidate predictors was based on the number of patients in the smallest group (return to sports group, or no return to sports group) [17]. Separate models were built for patient characteristics, trauma characteristics, baseline severity of knee complaints and MR findings. Candidate predictors with a univariate association of *P* <0.2 were all entered into a multivariable logistic regression analysis in one block (enter method). In the case of multicollinearity (r.0.5) of the candidate predictors, the variable with the strongest association (odds ratio; OR) with no return to sports was selected for the multivariable logistic regression analysis. In the latter analyses, variables with *P* > 0.2 were removed.

Variables with an association of *P* <0.2 in the multivariable logistic regression analysis of the separate models were selected for a final multivariable logistic regression analysis (enter method) with a combination of patient characteristics, trauma characteristics and baseline severity of knee complaints. Finally, the MR findings were added to the combined model to assess the additive value of an MR scan. A receiver operating characteristic curve was created and the area under the curve (AUC) was calculated to compare the separate models [18]. SPSS version 21.0 (SPSS Inc., Armonk, NY, USA) was used for all analyses.

## Results

### Patient inclusion

**Figure 1.**
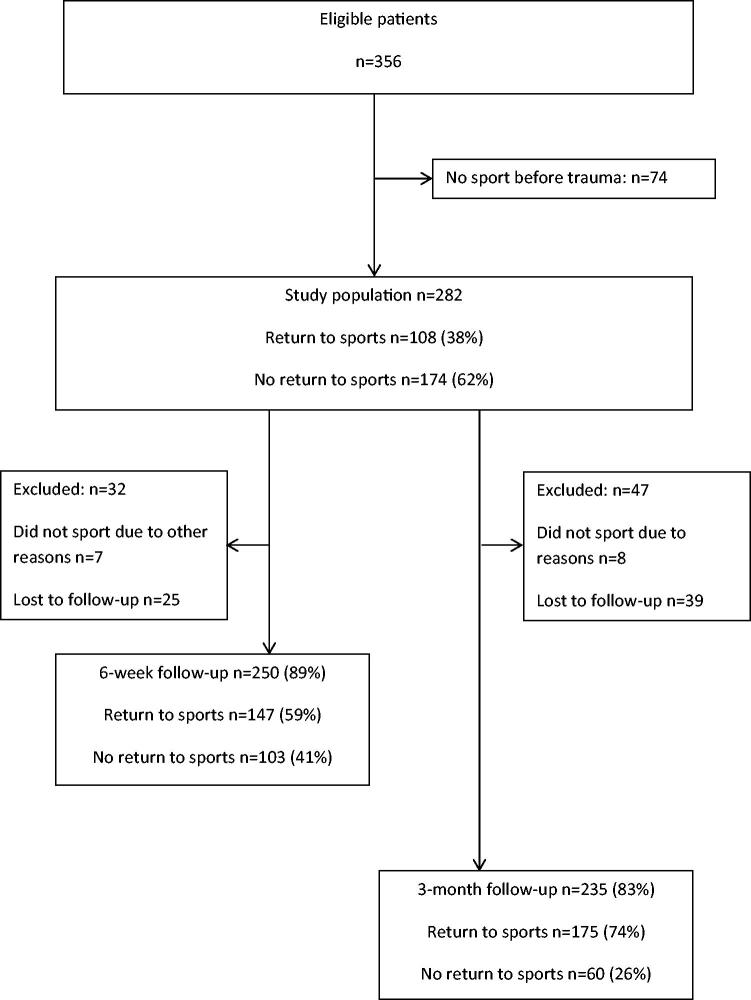
Flow chart of the process.

Figure 1 is a flow chart of the process. In the TACKLE trial, 836 patients were invited to participate. Of the 356 patients included in the RCT, 282 (79%) participated in sports before the knee trauma and were included in the present study. At six weeks and three months follow-up, 250 (89%) and 235 (83%) patients, respectively, were available for analysis.

### Baseline characteristics

Baseline characteristics are presented in Table 1. Median age was 32 (IQR: 26–39) years and 63% of the patients were male. Median time from trauma to study inclusion was 39 (IQR: 13–80) days. The four most commonly performed sports before trauma were: (i) soccer, (ii) fitness training or aerobics, (iii) athletics or running, and (iv) combat sport with 99 (35%), 60 (21%), 40 (14%) and 16 (6%) patients, respectively. For 188 (67%) patients the trauma occurred during sports, and in total, 114 (40%) patients experienced a rotational trauma.

**Table 1.** Baseline characteristics of included patients (*n* = 282).

	Study population
**Patient characteristics**	
Age in years, median (IQR)	32 (26–39)
Male gender	178 (63%)
Body mass index, median (IQR)	24.6 (22.7–26.9)
High educational level	117 (41%)
Musculoskeletal comorbidities	63 (22%)
Previous knee complaints	116 (41%)
Time from trauma to study inclusion in days, median (IQR)	39 (13–80)
Symptom on the right knee	128 (45%)
Sports before trauma	282 (100%)
Hours spent on sport per week, median (IQR)	3 (2–5)
Ball sport	126 (45%)
Sport in competition	124 (44%)
Soccer	99 (35%)
Fitness training/aerobics	60 (21%)
Athletics/running	40 (14%)
Combat sport	16 (6%)
Paid job before trauma	252 (89%)
Hours spent on paid job per week, median (IQR)	38 (30–40)
**Trauma characteristics**	
Occasion of trauma	
During sports	188 (67%)
During work	18 (6%)
At home	12 (4%)
During traffic	24 (9%)
Other	40 (14%)
Mechanism of trauma	
Fall	72 (26%)
Rotation	114 (40%)
Bump	19 (7%)
Squatting	24 (9%)
Other	51 (18%)
Immediate pain	208 (74%)
Immediate effusion	72 (26%)
Continuation activity impossible	196 (70%)
Popping sensation during trauma	89 (35%)
**Severity of knee complaints**	
Invited afterward consultation	106 (38%)
Severity of knee pain (NPRS previous 48 h), median (IQR)	3 (5–7)
Symptoms and limitations in activities (Lysholm), median (IQR)	73 (56–85)
Workload and sport participation (Tegnér), median (IQR)	3 (2–4)
Fear of pain, movement and injury (TSK-11), median (IQR)	26 (22–30)
KOOS pain, median (IQR)	58.3 (44.4–75)
KOOS symptoms, median (IQR)	64.3 (46.4–78.6)
KOOS function in daily living, median (IQR)	69.1 (50–85.7)
KOOS sport and recreation, median (IQR)	30 (15–55)
KOOS quality of life, median (IQR)	43.8 (37.5–50)

Data are presented as numbers (percentages), unless otherwise stated. Missing values ranged up to 0.7%. NPRS, numeric pain rating scale with scores from 0–10, with a higher score indicating more pain. Lysholm scale scored from 0–100, with a higher score indicating fewer problems. TSK-11: Shortened version of the Tampa scale for kinesiophobia scored from 11 to 44, with a higher score indicating more kinesiophobia. KOOS, knee injury and osteoarthritis outcome score ranging from 0 to 100, with a higher score indicating fewer problems. IQR: interquartile range.

Of the 282 patients included at baseline, 138 (49%) had received an MR scan (Table 2). Median time from trauma to MR scan was 48 (IQR: 23–88) days. In 114 (83%) patients, one or more abnormalities were detected on the MR scan. In 50 (36%) patients, there was a bone bruise of the FTJ and in 11 (8%) there was a (micro) fracture; also 25 (18%) patients had a traumatic (not horizontal) meniscal tear, 24 (17%) had an MCL/LCL distortion, 34 (25%) had an ACL/PCL tear, and 31 (22%) patients had cartilage defect.

**Table 2.** Knee MR findings of the 138 patients with the MR scan.

Findings on MR scan	Study population
Time from trauma to MR scan in days, median (IQR)	48 (23–88)
Abnormalities present	114 (83%)
Effusion	58 (42%)
Bone bruise FTJ	50 (36%)
(Micro) fracture	11 (8%)
Traumatic meniscal tear^a^	25 (18%)
MCL/LCL distortion^b^	24 (17%)
ACL/PCL tear^c^	34 (25%)
Cartilage damage^d^	31 (22%)
Combinations	
ACL/PCL tear and bone bruise FTJ	24 (17%)
Traumatic meniscal tear and bone bruise FTJ	13 (9%)
Traumatic meniscal tear and ACL/PCL tear	12 (9%)
MCL/LCL distortion and bone bruise FTJ	11 (8%)

Data are presented as numbers (percentages), unless otherwise stated. Missing values ranged up to 1.4%.

MR: magnetic resonance; IQR: interquartile range; FTJ: femorotibial joint; MCL: medial collateral ligament; LCL: lateral collateral ligament; ACL: anterior cruciate ligament; PCL: posterior cruciate ligament.

a Longitudinal, radial or complex meniscal tear.

b Grade I–III.

c Partial or complete tear.

d Grade I–IV.

### Return to sports

At baseline, 108 (38%) patients returned to sport on the pre-injury level or an adapted level). At six weeks and three months follow-up 147 (59%) and 175 (74%) patients, respectively, returned to sports.

### Associations with no return to sports

The results of the bivariate logistic regression analyses for return to sports are presented in the Supplementary Material. The results of the multivariable logistic regression analyses for no return to sports are shown in Table 3.

**Table 3.** Multivariable logistic regression analysis for return to sports.

Six week follow-up (*n* = 250)			Three month follow-up (*n* = 235)		
	OR	95%CI		OR	95%CI
**Patient characteristics**			**Patient characteristics**		
Time from trauma to inclusion	1.00	0.99–1.01	Time from trauma to inclusion	1.00	0.99–1.01
Return to sports at baseline	0.04	0.01–0.10*	Return to sports at baseline	0.12	0.04–0.31*
Age	1.10	1.05–1.15*	Age	1.04	1.00–1.08**
MSK comorbidities	2.04	0.91–4.57**			
Ball sport before trauma	2.23	1.14–4.33*			
AUC = 0.85 (95%CI: 0.80–0.89). R^2^ = 0.46			AUC = 0.73 (95%CI: 0.66–0.80). R^2^ = 0.20		
**Trauma characteristics**			**Trauma characteristics**		
Time from trauma to inclusion	1.01	1.00–1.01	Time from trauma to inclusion	1.00	0.99–1.01
Return to sports at baseline	0.04	0.02–0.11*	Return to sports at baseline	0.12	0.04–0.33*
Trauma during sport	1.89	0.96–3.72**	Trauma during sport	2.50	1.16–5.39*
Rotational trauma	1.64	0.85–3.16**	Rotational trauma	1.84	0.92–3.69**
Popping sensation	2.11	1.07–4.14*	Popping sensation	1.97	1.00–3.91*
AUC = 0.84 (95%CI: 0.79–0.89). R^2^ = 0.43			AUC = 0.78 (95%CI: 0.71–0.84). R^2^ = 0.27		
**Baseline severity of knee complaints**^a^			**Baseline severity of knee complaints**^b^		
Time from trauma to inclusion	1.00	1.00–1.01	Time from trauma to inclusion	1.00	0.99–1.01
Return to sports at baseline	0.09	0.04–0.24*	Return to sports at baseline	0.25	0.09–0.68*
Effusion previous week	1.83	0.93–3.62**	Effusion previous week	2.49	1.14–5.41*
NPRS previous 48 h	1.26	1.08–1.48*	NPRS previous 48 h	1.30	1.09–1.55*
Tegnér score	0.89	0.76–1.05**	KOOS QoL	0.97	0.94–1.00**
AUC = 0.83 (95% CI 0.78–0.89). R^2^ = 0.44			AUC = 0.81 (95% CI 0.75–0.87). R^2^ = 0.32		
**MR subgroup (*n* = 128)**			**MR subgroup (*n* = 121)**		
**Findings on MR scan**^c^			**Findings on MR scan**^d^		
Time from trauma to inclusion	1.00	0.99–1.01	Time from trauma to inclusion	1.01	1.00–1.02
Return to sports at baseline	0.08	0.02–0.27*	Return to sports at baseline	0.14	0.04–0.53*
Effusion	2.55	1.07–6.09*	Effusion	2.71	1.02–7.21*
Traumatic meniscal tear	2.17	0.71–6.66**	Traumatic meniscal tear	3.10	1.01–9.49*
			MCL/LCL distortion	0.35	0.10–1.26**
AUC = 0.80 (95%CI: 0.72–0.87). R^2^ = 0.37			AUC = 0.79 (95%CI: 0.70–0.88). R^2^ = 0.29		

Adjusted for time from trauma to inclusion and baseline return to sports. Missing values ranged up to 1.6%.

MR: magnetic resonance; 95%CI: 95% confidence interval; OR: odds ratio; AUC: area under the curve; MSK: musculoskeletal; NPRS: numeric pain rating scale on a scale from 0 to 10, with a higher score indicating more pain; KOOS: knee injury and osteoarthritis outcome score ranging from 0 to 100, with a higher score indicating fewer problems; QoL: quality of life; Tegnér score from 0 to 10, with a higher score indicating fewer problems. MCL/LCL distortion: distortion of the medial or lateral collateral ligament.

*P = <0.05. **P = <0.20.

a ‘KOOS QoL’ removed because of *P* > 0.2.

b ‘TSK-11’ removed because of *P* > 0.2.

c ^‘^BML FTJ’ and ‘fracture’ removed because of *P* > 0.2.

d ‘ACL/PCL tear’ removed because of *P* > 0.2.

*Patient characteristics.* At six weeks follow-up, ‘age,’ ‘musculoskeletal comorbidities’ and ‘ball sport before trauma’ predicted no return to sports with an AUC of 0.85 (95%CI:0.80–0.89). At three months follow-up, only ‘age’ predicted no return to sports with an AUC of 0.73 (95%CI: 0.66–0.80).

*Trauma characteristics.* At six weeks follow-up, ‘trauma during sport’, ‘rotational trauma’ and ‘popping sensation’ predicted no return to sports with an AUC of 0.84 (95%CI: 0.79–0.89). At three months follow-up, ‘trauma during sport’, ‘rotational trauma’ and ‘popping sensation’ predicted no return to sports with an AUC of 0.78 (95%CI: 0.71–0.85).

*Baseline severity of knee complaints.* At 6-weeks follow-up, ‘effusion during previous week,’ ‘NPRS previous 48 h’ and the ‘Tegnér score’ predicted no return to sports with an AUC of 0.83 (95%CI: 0.78–0.88). At three months follow-up ‘effusion during previous week,’ ‘NPRS previous 48 h’ and ‘KOOS QoL’ predicted no return to sports with an AUC of 0.81 (95%CI: 0.75–0.87).

*MR findings.* At six weeks follow-up, ‘effusion’ and ‘traumatic meniscal tear’ predicted no return to sports with an AUC of 0.80 (95%CI: 0.72–0.87). At three months follow-up, ‘effusion’, ‘traumatic meniscal tear’ and ‘MCL/LCL distortion’ predicted no return to sports with an AUC of 0.79 (95%CI: 0.70–0.87).

### Additive value of the MR scan

The results of the multivariable logistic regression analyses for return to sports of the combined models and the additive value of the MR scan are shown in Table 4.

**Table 4.** Multivariable logistic regression analysis of the combined models for return to sports.

	OR	95%CI		OR	95%CI
Six week follow-up (*n* = 250)			Three month follow-up (*n* = 235)		
Patient characteristics, trauma characteristics and baseline severity of knee complaints^a^			Patient characteristics, trauma characteristics and baseline severity of knee complaints^b^		
Time from trauma to inclusion	1.00	0.99–1.01	Time from trauma to inclusion	1.01	1.00–1.02
Return to sports at baseline	0.05	0.02–0.13*	Return to sports at baseline	0.19	0.07–0.52*
Age	1.09	1.04–1.14*	Trauma during sport	2.58	1.17–5.72*
NPRS previous 48 h	1.32	1.12–1.54*	Effusion previous week	2.77	1.27–6.05*
			NPRS previous 48 h	1.33	1.12–1.59*
AUC = 0.86 (95%CI: 0.81–0.90). R^2^ = 0.47			AUC = 0.82 (95%CI: 0.76–0.88). R^2^ = 0.33		
**MR subgroup (*n* = 128)**			**MR subgroup (*n* = 121)**		
Patient characteristics, trauma characteristics, baseline severity of knee complaints and MR findings^c^			Patient characteristics, trauma characteristics, baseline severity of knee complaints and MR findings^d^		
Time from trauma to inclusion	1.00	0.99–1.01	Time from trauma to inclusion	1.01	0.99–1.02
Return to sports at baseline	0.04	0.02–0.10*	Return to sports at baseline	0.23	0.06–0.83*
Age	1.09	1.04–1.14*	NPRS previous 48 h	1.29	1.04–1.61*
			Traumatic meniscal tear	5.43	1.77–16.62*
AUC = 0.79 (95%CI: 0.71–0.87). R^2^ = 0.36			AUC = 0.79 (95%CI: 0.70–0.88). R^2^ = 0.28		

Adjusted for time from trauma to inclusion and baseline return to sports. Missing values ranged up to 1.6%. NPRS, numeric pain rating scale on a scale from 0 to 10, with a higher score indicating more pain.

MR: magnetic resonance; 95%CI: 95% confidence interval; OR: odds ratio; AUC: area under the curve.

* *P* = <0.05.

a ‘Ball sport before trauma’, ‘trauma during sports’, ‘rotational trauma’, ‘popping sensation’ and ‘Tegner score’ removed because of *P* > 0.05.

b ’Age’, ‘MSK comorbidities’, ‘effusion during previous week’, ‘rotational trauma’, ‘popping sensation’ and ‘KOOS QoL’ removed because of *P* > 0.05.

c ‘NPRS previous 48h’, ‘effusion on MR scan’ and ‘traumatic meniscal tear’ removed because of *P* > 0.05.

d ’Trauma during sport’, ‘effusion previous week’, effusion on MR scan and ‘MCL/ LCL distortion’ removed because of *P* > 0.05.

Combining the model of patient characteristics, trauma characteristics and baseline severity of knee complaints, the AUC was 0.86 (95%CI: 0.81–0.90) at six weeks follow-up and 0.82 (95%CI: 0.76–0.88) at three months follow-up. When adding the MR information, the AUC was 0.79 (95%CI: 0.71–0.87) at six weeks follow-up and 0.79 (95%CI: 0.70–0.88) at three months follow-up.

## Discussion

### Main findings

This study shows that at six weeks follow-up, 41% of the patients aged 18–45 years with traumatic knee complaints reported not to have returned to sports. After three months, this proportion was 26%. A combination of patient and trauma characteristics and knee complaints predicted no return to sports with an AUC of 0.86 at six weeks and of 0.82 at three months follow-up. Adding MR findings did not improve the prediction of ‘no return to sports’ at six weeks or three months follow-up (AUC 0.79 at both time points).

### Comparison with literature

We found no studies focusing on return to sports in patients with traumatic knee complaints seen in general practice. In secondary care, in a pair-matched comparison of conservatively treated patients with ACL injuries versus ACL reconstruction, a return to sports rate of 68% was seen after one year in the conservative group; this percentage was not significantly different between the groups [19]. The rate is lower than the 74% found in our study at three months follow-up. However, our population included patients with all types of intra/extra-articular damage due to trauma, in which only 34 (34.5%) patients had an ACL/PCL tear. The return to sport percentages for patients with traumatic knee complaints after surgery are even lower: i.e. 55% of the patients returned to sports after ACL reconstruction and 61% after arthroscopic lateral meniscectomy [20,21].

In patients after ACL reconstruction, younger age, male gender, playing elite sport and having a positive psychological response favoured returning to the preinjury level of sport [20,22]. In our study, there was no association between the Tegnér score and the TAMPA scale with no return to sports. Possibly, these factors play an essential role in the return to pre-injury level of sports, but not in return to an adapted level of sports.

In this study, an MR scan had no additive value to patient/trauma characteristics and severity of knee complaints in predicting no return to sports at six weeks and three months follow-up. Possibly, an MR scan can be additive in revealing information regarding the underlying cause of the knee complaints, which can be important in a later stage, for example, in predicting re-injury. Our finding is, however, in accordance with a recent study on the absence of an additive value of an MR scan in the prediction of recovery in patients with low back pain in general practice [23].

### Strengths and limitations

In this study, the *P*-value for the selection of variables for the multivariable analysis was set at 0.2. This might have caused a type 1 error; however, the number of variables tested was limited in the ratio of one per 10 patients. The final model of patient characteristics, trauma characteristics and baseline severity of knee complaints was used in the subgroup of patients to assess the additive value of MR scan. Although we did not validate the model in the subgroup, the groups were based on randomization and there were no differences in patient characteristics between the groups (with the exception of the time from trauma to study inclusion, for which the analyses were adjusted: data not shown).

However, to our knowledge, this is the first study on return to sports in patients with traumatic knee complaints in general practice. The results emphasize the difficulty patients with traumatic knee complaints have with return to sports. Identification of important predictors for no return to sports may serve to improve the treatment of patients with traumatic knee complaints in general practice.

### Implications

Future research should focus on all potential biological, psychological and social factors influencing return to sports. A large observational cohort with long-term follow-up is needed to be able to give insight into which factors are associated with no return to sports on a pre-injury level.

Until then, based on the results of our study, the GP can use the information gathered during history taking on patient characteristics, trauma characteristics, and the amount of pain to inform patients about the odds of retuning to sports. Subsequently, the GP may consider referring patients at high-risk of no return to sports to physiotherapy. At six weeks, for patients who are older and have more pain the odds of return to sports decreases and at three months, for patients who experienced trauma during sport, who had effusion during the previous week and in patients with more pain the odds of return to sports decreases. However, the GP has to be aware that there may be other factors, which we have not measured that may contribute to no return to sports.

## Conclusion

This study shows that at six weeks follow-up, two-fifths of the patients aged 18–45 years with traumatic knee complaints reported not to have returned to sport. After three months, this proportion was one in four. A combination of patient and trauma characteristics and knee complaints predicted ‘no return to sports’ well. MR findings had no additive predictive value.

## Supplementary Material

Results of the bivariate logistic regression analysis for return to sports
