# Supplementary material for: Predicting no return to sports after three months in patients with traumatic knee complaints in general practice by combining patient characteristics, trauma characteristics and knee complaints
Source: Eur J Gen Pract. 2019 Aug 21;25(4):205–13. doi: 10.1080/13814788.2019.1646241 (PMC6853237; doi:10.1080/13814788.2019.1646241)
Supplement: Results of the bivariate logistic regression analysis for return to sports [file IGEN_A_1646241_SM1787.docx]

| Appendix. Results of the bivariate logistic regression analysis for return to sports | | | | | | | |
| --- | --- | --- | --- | --- | --- | --- | --- |
|  | **6-weeks follow-up** | | | **3-months follow-up** | | | |
|  | **Return**  **(*n* = 147)** | **No return**  **(*n* = 103)** | **OR (95% CI)** | **Return**  **(*n* = 175)** | | **No return**  **(*n* = 60)** | **OR (95% CI)** |
| **Patient characteristics^1^** |  |  |  |  | |  |  |
| Age in years, median (IQR) | 32 (25, 39) | 34 (27, 41) | 1.09 (1.04-1.14)* | 33 (25, 39) | | 32 (27, 40.8) | 1.04 (1.00-1.08)** |
| Male gender | 92 (62.6%) | 66 (64.1%) | 0.88 (0.47-1.65) | 110 (62.9%) | | 39 (65%) | 0.85 (0.44-1.64) |
| BMI, median (IQR) | 24.7 (22.6, 26.9) | 24.7 (22.9, 27) | 1.04 (0.96-1.14) | 24.6 (22.8, 26.6) | | 24.2 (22.1, 26.9) | 1.01 (0.92-1.10) |
| High educational level | 67 (45.6%) | 41 (39.8%) | 1.21 (0.65-2.25) | 76 (43.4%) | | 28 (46.7%) | 1.45 (0.77-2.75) |
| MSK comorbidities | 29 (19.7%) | 27 (26.2%) | 1.80 (0.84-3.86)** | 40 (22.9%) | | 14 (23.3%) | 1.01 (0.47-2.17) |
| Previous knee complaints | 57 (38.8%) | 47 (45.6%) | 1.14 (0.77-2.69) | 69 (39.4%) | | 29 (48.3%) | 1.38 (0.72-2.61) |
| Ball sport before trauma | 58 (39.5%) | 56 (54.4%) | 1.56 (0.85-2.87)** | 76 (43.4%) | | 33 (55%) | 1.24 (0.65-2.35) |
| Hours sport p/w, median (IQR) | 3 (2, 5) | 3 (2, 5) | 0.97 (0.87-1.10) | 3 (2, 5) | | 4 (2, 5) | 1.03 (0.93-1.15) |
| Paid job before trauma | 130 (88.4%) | 95 (92.2%) | 0.78 (0.24-2.52) | 157 (89.7%) | | 55 (91.7%) | 0.84 (0.24-2.96) |
| Hours spend on paid job p/w, median (IQR) | 36 (30, 40) | 40 (32, 40) | 1.01 (0.98-1.04) | 40 (32, 40) | | 38 (28, 40) | 0.98 (0.95-1.02) |
| **Trauma characteristics^2^** |  |  |  |  | |  |  |
| Trauma during sport | 93 (63.3%) | 74 (71.8%) | 1.92 (1.00-3.71)* | 110 (62.9%) | | 47 (78.3%) | 2.45 (1.16-5.20)* |
| Rotational trauma | 48 (32.7%) | 56 (54.4%) | 2.01 (1.08-3.74)* | 64 (36.6%) | | 37 (61.7%) | 2.15 (1.12-4.14)* |
| Immediate pain | 106 (27.1%) | 78 (75.7%) | 1.06 (0.53-2.11) | 127 (72.6%) | | 47 (78.3%) | 1.21 (0.57-2.55) |
| Immediate effusion | 36 (24.5%) | 31 (30.1%) | 1.09 (0.56-2.12) | 42 (24%) | | 18 (30%) | 1.22 (0.61-2.44) |
| Continuation activity impossible | 94 (63.9%) | 77 (74.8%) | 1.00 (0.50-1.99) | 120 (68.6%) | | 45 (75%) | 0.83 (0.39-1.74) |
| Popping sensation | 38 (25.9%) | 48 (46.6%) | 2.42 (1.27-4.59)* | 52 (29.7%) | | 31 (51.7%) | 2.32 (1.22-4.41)* |
| **Severity of knee complaints^3^** |  |  |  |  | |  |  |
| Invited afterward consultation | 69 (46.9%) | 29 (28.2%) | 0.96 (0.45-2.05) | 68 (38.9%) | | 18 (30%) | 1.41 (0.63-3.15) |
| Effusion previous week | 56 (38.1%) | 73 (70.9%) | 2.18 (1.13-4.20)* | 74 (42.3%) | | 46 (76.7%) | 3.20 (1.53-6.71)* |
| NPRS previous 48h, median (IQR) | 5 (2, 6) | 6 (4, 7) | 1.29 (1.11-1.50)* | 4 (2, 6) | | 6 (5, 7) | 1.36 (1.15-1.60)* |
| Tegner score, median (IQR) | 4 (3, 5) | 2 (1, 4) | 0.87 (0.75-1.03)** | 3 (2, 5) | | 2 (1, 4) | 0.96 (0.82-1.13) |
| TSK-11, median (IQR) | 25 (21, 29) | 27 (24, 32) | 1.04 (0.98-1.10) | 25 (21, 28) | | 28 (24, 32) | 1.05 (0.99-1.12)** |
| KOOS QoL, median (IQR) | 50 (37.5, 56.2) | 37.5 (31.3, 50) | 0.98 (0.95-1.01)** | 50 (37.5, 56.3) | | 37.5 (31.3, 43.8) | 0.96 (0.92-0.99)* |
|  |  | | |  | | | |
|  | **Return**  **(*n* = 73)** | **No return**  **(*n* = 55)** | **OR (95% CI)** | **Return**  **(*n* = 85)** | **No return**  **(*n* = 36)** | | **OR (95% CI)** |
| **Magnetic Resonance findings** |  |  |  |  |  | |  |
| Effusion | 21 (28.8%) | 33 (60%) | 2.83 (1.20-6.65)* | 28 (32.6%) | 22 (61.1%) | | 3.03 (1.23-7.51)* |
| Bone bruise FTJ | 17 (23.3%) | 31 (56.4%) | 2.48 (1.05-5.84)* | 28 (32.6%) | 17 (47.2%) | | 1.11 (0.46-2.67) |
| Fracture | 2 (2.7%) | 9 (16.4%) | 3.42 (0.68-17.23)** | 5 (5.8%) | 5 13.9%) | | 1.79 (0.46-6.89) |
| Traumatic meniscal tear | 8 (11%) | 14 (25.5%) | 2.65 (0.88-8.03)** | 8 (9.3%) | 12 (33.3%) | | 4.79 (1.65-13.91)* |
| MCL/ PCL distortion | 11 (15.1%) | 12 (21.8%) | 0.92 (0.34-2.50) | 18 (20.9%) | 4 (11.1%) | | 0.32 (0.10-1.08)** |
| ACL/ PCL tear | 12 (16.4%) | 20 (36.4%) | 2.05 (0.81-5.20) | 16 (18.6%) | 13 (36.1%) | | 1.96 (0.78-4.92)** |
| Cartilage damage | 15 (20.5%) | 15 (27.3%) | 1.07 (0.42-2.72) | 17 (19.8%) | 10 (27.8%) | | 1.26 (0.48-3.31) |
| Adjusted for duration of complaints at study inclusion and return to sports at baseline. Data is presented as numbers (percentages) unless otherwise stated. Missing values ranged up to 1.6%. p/ w: per week. MR: magnetic resonance. IQR: inter quartile range. 95% CI; 95% confidence interval. OR: odds ratio. BMI: body mass index. MSK: musculoskeletal. NPRS: Numeric Pain Rating Scale on a scale from 0 to 10, with a higher score indicating more pain. TSK-11: Shortened version of the Tampa scale for kinesiophobia, from 11 to 44, with a higher score indicating more kinesiophobia. KOOS: Knee injury and Osteoarthritis Outcome Score on a scale from 0 to 100, with a higher score indicating less problems. QoL: quality of life. Tegner score from 0 to 10, with a higher score indicating less problems. FTJ: femorotibial joint. MCL: medial collateral ligament. LCL: lateral collateral ligament. ACL: anterior cruciate ligament. PCL: posterior cruciate ligament. **P* = < 0.05. ***P* = < 0.20. ^1^The variables ‘sports in competition’ and the ‘Tegner score before trauma’ were removed from the analysis because of multicollinearity with ball sport before trauma. ^2^The variable ‘trauma during ball sport’ was removed from the analyses because of multicollinearity with trauma during sport. ^3^The variables ‘pain during previous week’, the ‘Lysholm score’, ‘KOOS pain’, ‘KOOS symptoms’ and ‘KOOS function in daily living’ and KOOS sport and recreation were removed from the analyses because of multicollinearity. | | | | | | | |
